# *NDRG2* mRNA levels and miR-28-5p and miR-650 activity in chronic lymphocytic leukemia

**DOI:** 10.1186/s12885-018-4915-3

**Published:** 2018-10-22

**Authors:** Yu-Qiong Yang, Tian Tian, Hua-Yuan Zhu, Jin-Hua Liang, Wei Wu, Jia-Zhu Wu, Yi Xia, Li Wang, Lei Fan, Jian-Yong Li, Wei Xu

**Affiliations:** 1 Department of Hematology, the First Affiliated Hospital of Nanjing Medical University, Province Hospital, Nanjing, 210029 Jiangsu China; 2 Key Laboratory of Hematology of Nanjing Medical University, Nanjing, 210029 China; 3 Collaborative Innovation Center for Cancer Personalized Medicine, Nanjing, 210029 China

**Keywords:** Chronic lymphocytic leukemia, *NDRG2*, miR-28-5p, miR-650

## Abstract

**Background:**

*NDRG2* is identified as a tumor suppressor gene in many tumors, and functions in cell proliferation, differentiation and apoptosis. Recent data indicate that *NDRG2* expression is up-regulated by *TP53.* Moreover, proposed mechanisms of *NDRG2* inactivation include epigenetic silencing of the *NDRG2* promoter and down-regulation by microRNAs (miRNAs). However, few studies have ever been done on the role of *NDRG2* and the *NDRG2*-regulating miRNAs interference in chronic lymphocytic leukemia (CLL).

**Methods:**

*NDRG2* and microRNAs mRNA levels in CLL subjects were assessed by quantitative real-time polymerase chain reaction (qRT-PCR). The dual-luciferase reporter assay was performed to determine NDRG2-related miRNAs. Low expression of mature exogenous miRNAs in CLL cells was established by transient transfection. NDRG2 protein levels in CLL cells were detected by western blot. In addition, flow cytometry was conducted to examine the apoptosis of CLL cells.

**Results:**

Lower expression of *NDRG2* was found in the B-cells from 102 CLL patients compared the 40 normal subjects (*P* < 0.001). Patients with advanced Binet stage (*P* = 0.001), high lactate dehydrogenase (LDH) level (*P* = 0.036), un-mutated immunoglobulin heavy chain variable region gene (IGHV) (*P* = 0.004) and those with p53 aberrations (*P* < 0.001) had a markedly lower levels of *NDRG2* mRNA. This decrease was associated with briefer time-to-treatment (*P* = 0.001) and poorer survival (*P* < 0.001). High expression of miR-28-5p and miR-650 was associated with Binet B/C stage (*P* = 0.044) and IGHV un-mutated (*P* = 0.011), as well as Binet B/C stage (*P* = 0.013) and p53 aberrations (*P* = 0.037), respectively. Inhibition of miR-28-5p or miR-650 could induce more apoptosis in CLL cells with germline *TP53*.

**Conclusions:**

*NDRG2* mRNA levels might be a useful prognostic variable for patients of CLL and up-regulating *NDRG2* transcription may be a therapy approach in CLL without p53 aberrations.

**Electronic supplementary material:**

The online version of this article (10.1186/s12885-018-4915-3) contains supplementary material, which is available to authorized users.

## Background

*NMYC* downstream-regulated gene-2 (*NDRG2*) serves as a tumor suppressor gene, and play essential parts in cell proliferation, differentiation and apoptosis. Associations between *NDRG2* expression and cancer biology were described in brain, liver, lung, thyroid and breast cancers as well as in hematologic neoplasms [1–6]. Recent data indicate that *NDRG2* expression, up-regulated by *TP53* [7]*,* can inhibit *STAT3* activation and NF-κB activity [8, 9]. Proposed mechanisms of *NDRG2* inactivation include epigenetic silencing of the *NDRG2* promoter and down-regulation by microRNAs (miRNAs) [1, 10]. However, few data are available on the role of *NDRG2* in chronic lymphocytic leukemia (CLL).

## Methods

### Subjects

Consecutive 102 CLL patients untreated were enrolled between January 2004 and December 2013. All subjects were given written informed consent complying the requirements of the Declaration of Helsinki. The research was approved by the Institutional Review Boards of Nanjing Medical University. CLL diagnosis was based on the criteria specified in the National Cancer Institution [11]. Variables ascertained in diagnosis included age, gender, Binet staging, blood absolute lymphocyte count (ALC), lactate dehydrogenase (LDH), β_2_-microglobulin (β_2_-MG), CD38, ZAP-70, mutation status of IGHV gene, *TP53* mutation (exon 4–9) status, and cytogenetics (studied by fluorescence in situ hybridization; FISH). In addition, 40 age-matched healthy controls (HC) were recruited between January 2012 and December 2013 from the center of Health Examination.

### Primary cell cultures

CLL cells from 11 subjects were isolated from venous blood by density-gradient centrifugation. > 90% of cells were B-cells, as determined by staining for CD5^+^CD19^+^ coexpression (Becton Dickinson, USA), were cultured in RPMI-1640 media. Ten percent fetal calf serum (Gibco, USA) was added to the media. CLL cells were cultured at 37 °C with 5% CO_2_ in humidified atmosphere.

### *NDRG2* mRNA detection

Whole RNAs was extracted using Trizol reagent (Invitrogen). RNA was reversely transcribed using random hexamers, and amplified with fluorescent dye SYBR Green MasterMix and qRT-PCR specific reverse primers (Additional file 1). β-actin was used as an internal control. Reaction conditions for *NDRG2* and β-actin were one cycle at 95 °C for 5 min followed by 35 cycles at 95 °C for 30 s, 60 °C for 30 s and 72 °C for 30 s. A final extention was run at 72 °C for 5 min. Relative expression was analyzed using the comparative cycle threshold (Ct) method. ΔCt was calculated by subtracting the Ct of β-actin from those for the gene of interest. Relative amounts of the interest gene were calculated using eq. 2^-Δ Ct^. Reactions for qRT-PCR were performed in triplicate, using the Applied Biosystems ABI 7500 Real-time PCR system (Applied Biosystems, USA). Sequences of amplifed production were confirmed via DNA sequencing.

### Dual luciferase reporter assay

*NDRG2*-related miRNAs were initially identified in bioinformatics miRNA databases such as target scan (http:/www.targetscan.org/) and miR-Base (http://www.mirbase.org/). Dual luciferase reporter assay (Promega, USA) was used to evaluate whether the conserved miRNAs with higher scores binds directly to *NDRG2*. Renilla-luciferase assay was performed with a modified expression pEZX vector containing the complete 3′ untranslated regions (UTR) region of *NDRG2* cloned in the 3’UTR region of dual luciferase gene. The miRNAs or negative controls (NC) were transfected in the HEK293T cell line together with pEZX vector using lipofectamine 2000 for dual-luciferase assay. HEK293T cell line was obtained from ATCC (American Type Culture Collection, Manassas, VA, USA, ATCC@-ACS 4500) in 2012. The cell line has been authenticated by using Single Tandem Repeat (STR) profiling method and there is no mycoplasma contamination. All cells were transfected for 24 h and assayed using a Luciferase Assay Kit (Promega, USA).

### Transient transfection

CLL cells were transferred in 6-well plates by density of 5.0 × 10^6^ cells/well and transiently transfected with 100 nM of mature miRNA inhibitors of miR-28-5p and miR-650 and 100 nM as random negative-control miRNA (miR-NC) (GenePharma Company, China) using Lipofectamine 2000 Transfection Reagent (Invitrogen, USA) according to the manufacturer’s protocol.

### Western blotting

Cells were harvested with lysis buffer 24 h after transfection with miRNA inhibitors (including miR-28-5p, miR-650 and negative control miRNA inhibitors). Protein concentration was calculated using BCA (Beyotime, China). Total protein was separated using 10% sodium dodecyl sulfate–polyacrylamide gel, transferred to PVDF membranes and incubated with goat monoclonal antibody against *NDRG2* (Santa Cruz Biotechnology, USA) in 1:200 dilutions. The secondary antibody was rabbit anti-goat IgG (Santa Cruz Biotechnology, USA) in 1:2500 dilutions. The normalized control used was GAPDH.

### Apoptosis assay

The apoptotic ratio of CLL cells was detected using Annexin V/propidium iodide (PI) flow cytometric assay (Becton, Dickinson and Company). CLL cells after 24 h-transfection were washed twice with cold phosphate-buffered saline (PBS), and then re-suspended in 500 μL binding buffer (Bestbio, Shanghai, China). Annexin V and PI were added to the transferred cells and the plate was incubated in the dark for 15 min at room temperature. Apoptosis cells analysis was performed using a FACSCalibur flow cytometer and CellQuest software (BD Biosciences, USA).

### Statistical analyses

Statistical analyses were performed using SPSS software for Windows (version 20.0). The difference of target gene mRNA expression between groups with different prognostic factors was described using the Mann–Whitney U test. The difference of miRNA and *NDRG2* expression and apoptosis rate between groups was calculated using the Paired-Samples t- Test. Survival was calculated as time from diagnosis until death or loss to follow-up or to May 2017. Time to first treatment (TTT) was calculated as interval from diagnosis until first CLL-specific treatment or the last follow-up. Survival and TTT were estimated by the Kaplan-Meier method and results were compared using the log-rank test. Prognostic influence of variables was tested using the Cox proportional hazards model in univariate and multivariate analyses. Protein bands of Western blot were quantified through the Image J program for Windows after normalizing the data for GAPDH. 2-sided *P*-values < 0.05 were considered statistically significant.

## Results

### Clinical variables

Baseline variables for subjects are shown in Table 1. Median age at diagnosis was 59 years (range, 16–86 years). Sixty-nine were males. Thirty-eight subjects (37.2%) were in Binet A, 33 (32.4%) in Binet B and 31 (30.4%) in Binet C. p53 aberrations including *TP53* mutation or p53 deletion was detected in 24 patients (23.5%). Median duration of follow-up is 71 months (range, 14–160 months). Sixty-five received chemotherapy or chemoimmunotherapy, and the rest patients were untreated complying with the international workshop on chronic lymphocytic leukemia (IWCLL) criteria. Twenty-five subjects died of CLL-related causes.

**Table 1:** Clinical and biological variables of 102 subjects with chronic lymphocytic leukemia

Characteristic	Value (%)
Age	
≤ 60 years	58 (56.9)
> 60 years	44 (43.1)
Sex	
Male	69 (67.6)
Female	33 (32.4)
Binet stage	
A	38 (37.2)
B	33 (32.4)
C	31 (30.4)
Lymphocytes	
< 50 × 10^9^/L	71 (69.6)
≥ 50 × 10^9^/L	31 (30.4)
LDH	
≤ ULN	79 (77.5)
> ULN	20 (22.5)
β_2_-MG	
≤ ULN	34 (33.3)
> ULN	68 (66.7)
CD38 (*n* = 100)	
< 30%	75 (75.0)
≥ 30%	25 (25.0)
ZAP-70 (*n* = 100)	
< 20%	55 (55.0)
≥ 20%	45 (45.0)
IGHV (*n* = 101)	
Mutated	61 (60.4)
Unmutated	40 (39.6)
p53 aberrations	
No *TP53* mutation or p53 deletion	78 (76.4)
*TP53* mutation or p53 deletion	24 (23.5)
del (11q22.3) (*n* = 93)	
Negative	78 (83.9)
Positive	15 (16.1)
del (13q14) (*n* = 65)	
Negative	39 (60.0)
Positive	26 (40.0)
+ 12 (*n* = 81)	
Negative	69 (85.2)
Positive	12 (14.8)

### *NDRG2* expression and association with other variables

**Fig. 1:**
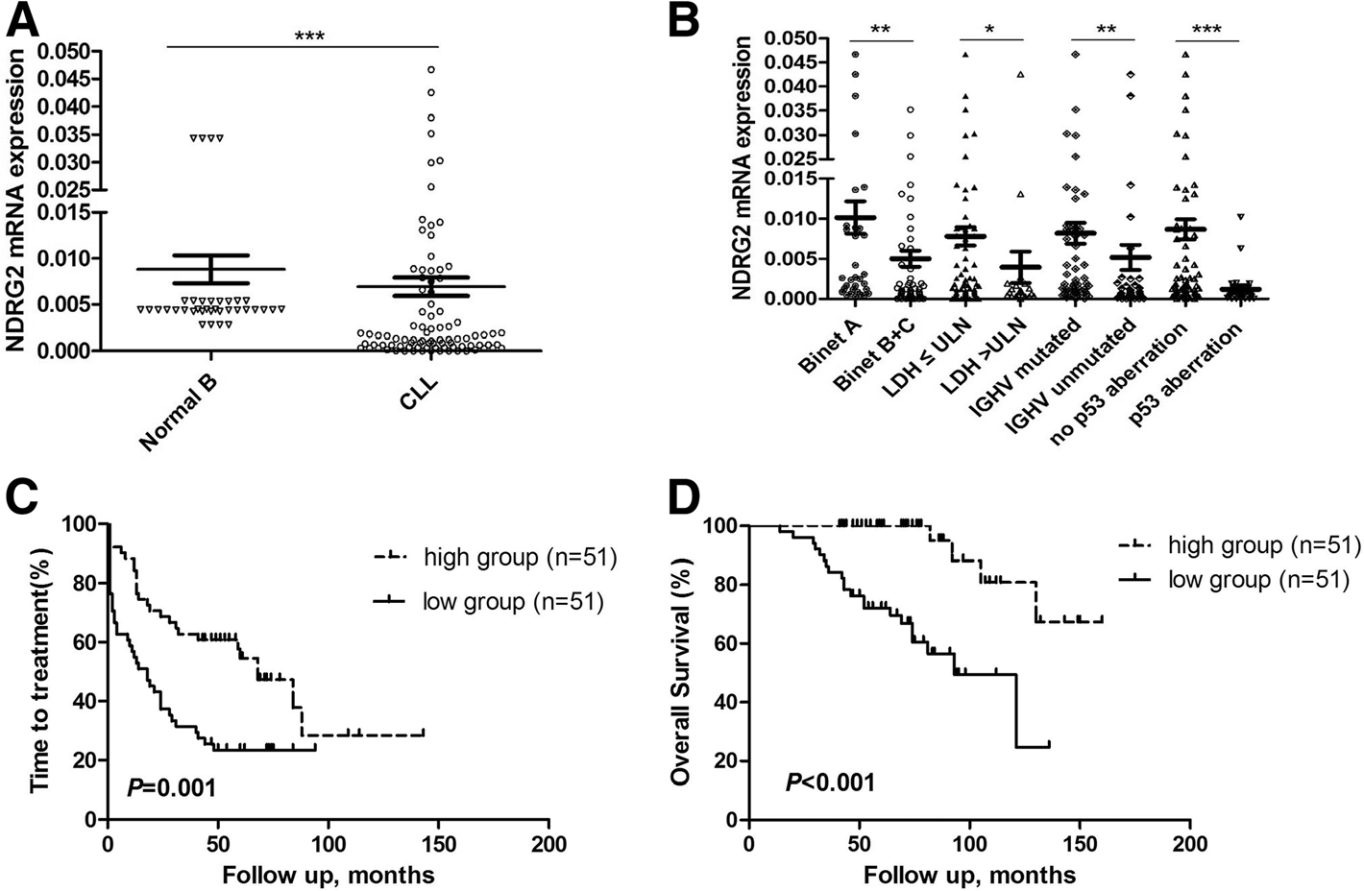
**a** Determination of *NDRG2* mRNA expression in the102 CLL patients and 40 normal B-cell samples by RT-PCR. ***P < 0.001. **b** Dot-plot of *NDRG2* mRNA expression in CLL patients. Significant associations were found with Binet stage B/C vs. A, high LDH level vs. normal LDH level, IGHV un-mutated vs. IGHV mutated and p53 aberrations vs. no p53 aberrations. ***P < 0.001, **P < 0.01, *P < 0.05. **c** Time to treatment (TTT) curve of CLL based on *NDRG2* mRNA by Kaplan-Meier estimation. **d** Overall survival (OS) curve of CLL based on *NDRG2* mRNA by Kaplan-Meier estimation

Relative expression level of *NDRG2* mRNA in subjects versus normal controls was 0 to 0.0467 (median: 0.0017) and from 0.0028 to 0.0343 (median: 0.0044). Down-expression of *NDRG2* was found in CLL patients compared to HC (*P* < 0.001, Fig. 1a).

*NDRG2* expression was analyzed in CLL patients for association with baseline variables (Table 2). Significant associations were found with Binet stage B/C vs. A (*P* = 0.001), high LDH level vs. normal LDH level (*P* = 0.036), *IGHV* un-mutated vs. IGHV mutated (*P* = 0.004) and p53 aberrations vs. no p53 aberrations (*P* < 0.001) (Fig. 1b).

**Table 2:** Associations between *NDRG2* mRNA and baseline variables for the 102 CLL subjects

	Median (95% CI)	*P*-value
Age (years)		
≤ 60 years	0.0015 (0–0.0303)	0.280
> 60 years	0.0017 (0–0.0340)	
Gender		
Male	0.0018 (0–0.0366)	0.194
Female	0.0009 (0–0.0193)	
Binet stage		
A	0.0036 (0.0004–0.0428)	0.001
B/C	0.0011 (0–0.0245)	
Lymphocytes (×10^9^/L)		
< 50	0.0018 (0–0.0238)	0.618
≥ 50	0.0013 (0–0.0398)	
LDH		
≤ ULN	0.0025 (0–0.0303)	0.036
> ULN	0.0008 (0–0.0375)	
β_2_-MG		
≤ ULN	0.0012 (0–0.0341)	0.539
> ULN	0.0018 (0–0.0330)	
CD38 (*n* = 100)		
< 30%	0.0020 (0–0.0358)	0.063
≥ 30%	0.0008 (0–0.0268)	
ZAP-70 (*n* = 100)		
< 20%	0.0017 (0.0002–0.0389)	0.244
≥ 20%	0.0020 (0–0.0274)	
IGHV (*n* = 101)		
Mutated	0.0031 (0.0002–0.0303)	0.004
Unmutated	0.0008 (0–0.0371)	
p53aberration		
No *TP53* mutation or p53 deletion	0.0027 (0.0001–0.0353)	< 0.001
*TP53* mutation or p53 deletion	0.0003 (0–0.0009)	

### *NDRG2* expression is associated with TTT and survival

Patients were subdivided into high- and low-*NDRG2* groups by *NDRG2* expression level (median split) to analyze the survival. The cohort of CLL was initially split into low (*N* = 51) and high (N = 51) based on a value of 0.0017. With the median follow-up of 71 months (range, 14–160 months), patients in the low *NDRG2* mRNA cohort had a briefer TTT (median, 18 months, range, 1–94 months, *P* = 0.001) (Fig. 1c) and worse survival (median, 93 months, range, 14–136 months, *P* < 0.001) (Fig. 1d) compared to those in high *NDRG2* mRNA cohort.

Univariate analysis indicated that Binet stage B/C, un-mutated IGHV, p53 aberrations and low *NDRG2* mRNA were significantly associated with a brief TTT (Table 3), whereas multivariate analysis only showed significance with Binet stage B/C (*P* = 0.008; HR 2.48; [1.27, 4.84]), p53 aberrations (*P* = 0.015; HR 2.46; [1.19, 5.09]) and low *NDRG2* mRNA (*P* = 0.022; HR 2.01; [1.11, 3.64]) (Table 3).

**Table 3:** Univariate and multivariate Cox regression analysis for time to treatment (TTT) and overall survival (OS) in 102 subjects with chronic lymphocytic leukemia

Variables	Univariate analysis (Kaplan-Meier)	Multivariate analysis	
	*P*-value	HR(95%CI)	*P*-value
TTT			
Age >60 y	0.842	0.83 (0.46–1.49)	0.530
Sex (male)	0.821	1.52 (0.85–2.71)	0.159
Binet B/C stage	<0.001	2.48 (1.27–4.84)	0.008
ALC ≥50×10^9^/L	0.977	0.90 (0.50–1.63)	0.721
LDH>ULN	0.767	0.72 (0.36–1.43)	0.348
β_2_-MG>ULN	0.095	1.49 (0.81–2.74)	0.204
CD38 (≥30%)	0.164	1.01 (0.55–1.84)	0.988
ZAP-70 (≥20%)	0.308	0.89 (0.50–1.59)	0.692
IGHV unmutated	<0.001	1.68 (0.97–2.92)	0.067
p53 aberration	<0.001	2.46 (1.19–5.09)	0.015
*NDRG2* expression (high)	0.001	2.01 (1.11–3.64)	0.022
OS			
Age >60 y	0.700	0.89 (0.34–2.35)	0.814
Sex (male)	0.633	1.33 (0.51–3.48)	0.563
Binet B/C stage	0.028	0.85 (0.22–3.33)	0.814
ALC ≥50×10^9^/L	0.524	0.70 (0.22–2.25)	0.549
LDH>ULN	0.071	1.81 (0.52–6.26)	0.349
β_2_-MG>ULN	0.813	1.29 (0.46–3.59)	0.628
CD38 (≥30%)	0.002	2.01 (0.79–5.13)	0.143
ZAP-70 (≥20%)	0.079	1.31 (0.44–3.89)	0.632
IGHV unmutated	<0.001	3.03 (1.16–7.95)	0.024
p53 aberration	<0.001	3.95 (1.34–11.67)	0.013
*NDRG2* expression (high)	<0.001	3.43 (1.06–11.14)	0.040

Univariate analysis for overall survival (OS) suggested that Binet stage B/C, CD38-positive, un-mutated IGHV, p53 aberrations and low *NDRG2* mRNA were significantly associated with worse survival (Table 3). However, only un-mutated IGHV (*P* = 0.024; HR 3.03; [1.16, 7.95]), p53 aberrations (*P* = 0.013; HR 3.95; [1.34, 11.67]) and low *NDRG2* mRNA (*P* = 0.040; HR 3.43; [1.06, 11.14]) remained significant by multivariate analysis.

### miR-28-5p and miR-650 directly targeted *NDRG2*

**Fig. 2:**
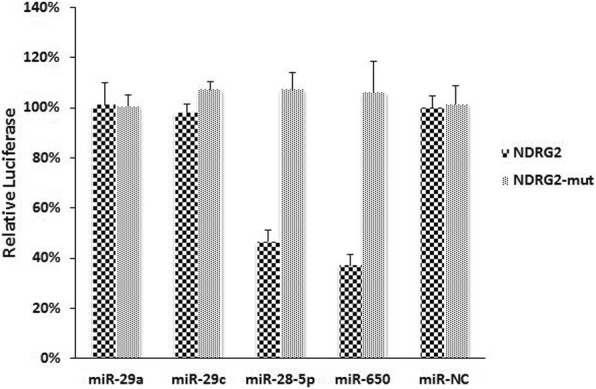
Dual luciferase assay performed in HEK293T cells suggested that miR-28-5p and miR-650 mimics significantly suppress the activity of *NDRG2* when compared to miR-NC

The computational algorithms, including TargetScan and RNAhydrid, were collectively used identify potential miRNAs that target *NDRG2*. As is shown in Additional file 2, the bioinformatics analysis identified four miRNAs (miR-29a, miR-29c, miR-28-5p and miR-650) as possible regulators of *NDRG2*. Moreover, we constructed luciferase reporter assays with 3′-untranslated regions (UTR) of *NDRG2* (pEZX-*NDRG2*–3’-UTR) to further determine whether *NDRG2* was directly regulated by these miRNAs. The luciferase reporter was introduced to 100 nM of these miRNAs mimics and miR-NC into HEK293T cells. The luciferase activity was significantly reduced in the cell line when pEZX-*NDRG2*–3’-UTR was transfected with miR-28-5p and miR-650 mimics. Furthermore, we introduced point mutations into the corresponding complementary sites in the 3’-UTR of *NDRG2* to eliminate the predicted miR-28-5p and miR-650 binding sites (mean percent of luciferase activity reduction in the two miRNAs of 47% and 37%, respectively, Fig. 2). The mutated luciferase reporter was unaffected by overexpression of miR-28-5p and miR-650. The results showed that miR-28-5p and miR-650 directly inhibited the expression of *NDRG2* by binding to the target sequence.

### miR-28-5p and miR-650 expression in CLL patients

qRT-PCR was performed in 30 CLL patients and 10 HC to detect the expression of miR-28-5p and miR-650. We found that miR-28-5p and miR-650 were upregulated in CLL cells compared with normal lymphocytes. Of the 30 patients and 10 HC, the relative level of miR-28-5p expression was from 1.1408 to 22.9426 (median: 4.8444) and from 1.1684 to 3.0863 (median: 2.2215) and the relative expression level of miR-650 spanned from 0.0060 to 1.7415 (median: 0.0811) and from 0.0037 to 0.1023 (median: 0.0191), respectively. A statistical significance was found between the CLL patients and HC in miR-28-5p (*P* < 0.001) and miR-650 (*P* = 0.006) expression. Then we analyzed the correlations between expression levels of the two miRNAs and patients’ clinical characteristics, including Binet stage, p53 aberrations, IGHV mutation status and ALC. A significant increase of miR-28-5p expression was observed in patients with Binet B/C (*P* = 0.044) and un-mutated IGHV (*P* = 0.011). Moreover, notable increase of miR-650 expression was observed in patients with Binet B/C (*P* = 0.013) and p53 aberrations (*P* = 0.037). *NDRG2* mRNA was detected in the 30 CLL cases aforementioned. Interestingly, we found that the expression of *NDRG2* was associated negatively with miR-28-5p (*r* = − 0.468, *P* = 0.009, Additional file 3) and miR-650 (*r* = − 0.411, *P* = 0.024, Additional file 3).

### miR-28-5p and miR-650 inhibitors up-regulated NDRG2 expression and induced CLL cells apoptosis

**Fig. 3:**
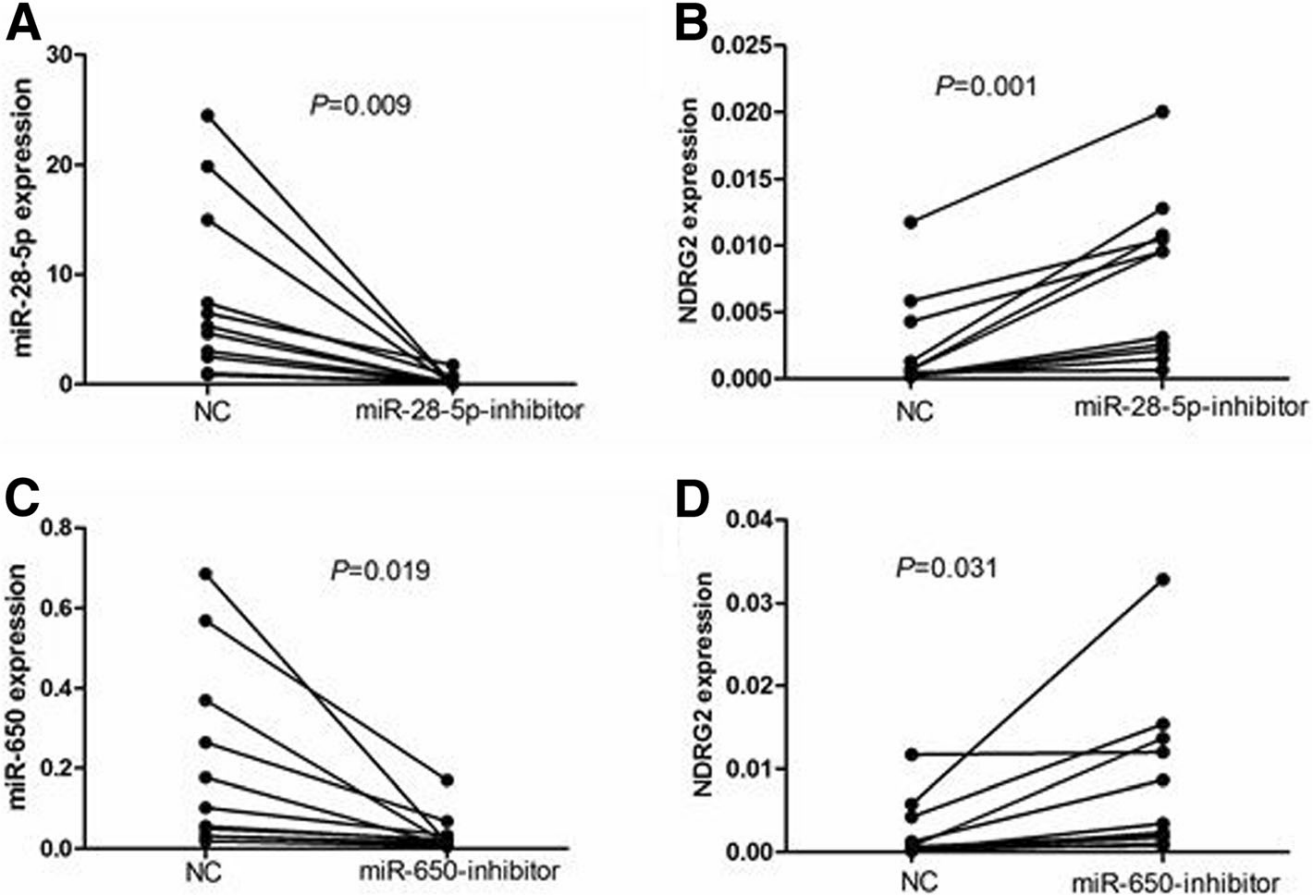
The expression of *NDRG2* and miRNAs before and after transfecting with miRNA inhibitors. The corresponding miRNAs fall significantly in groups treated with miR-28-5p and miR-650 inhibitors groups than treated with miR-NC (*P* = 0.009, **a**; *P* = 0.019, **c**), separately. The primary CLL cells express obviously increased *NDRG2* mRNA in miR-28-5p (*P* = 0.001) (**b**) and miR-650 (*P* = 0.031) (**d**) inhibitors groups

**Fig. 4:**
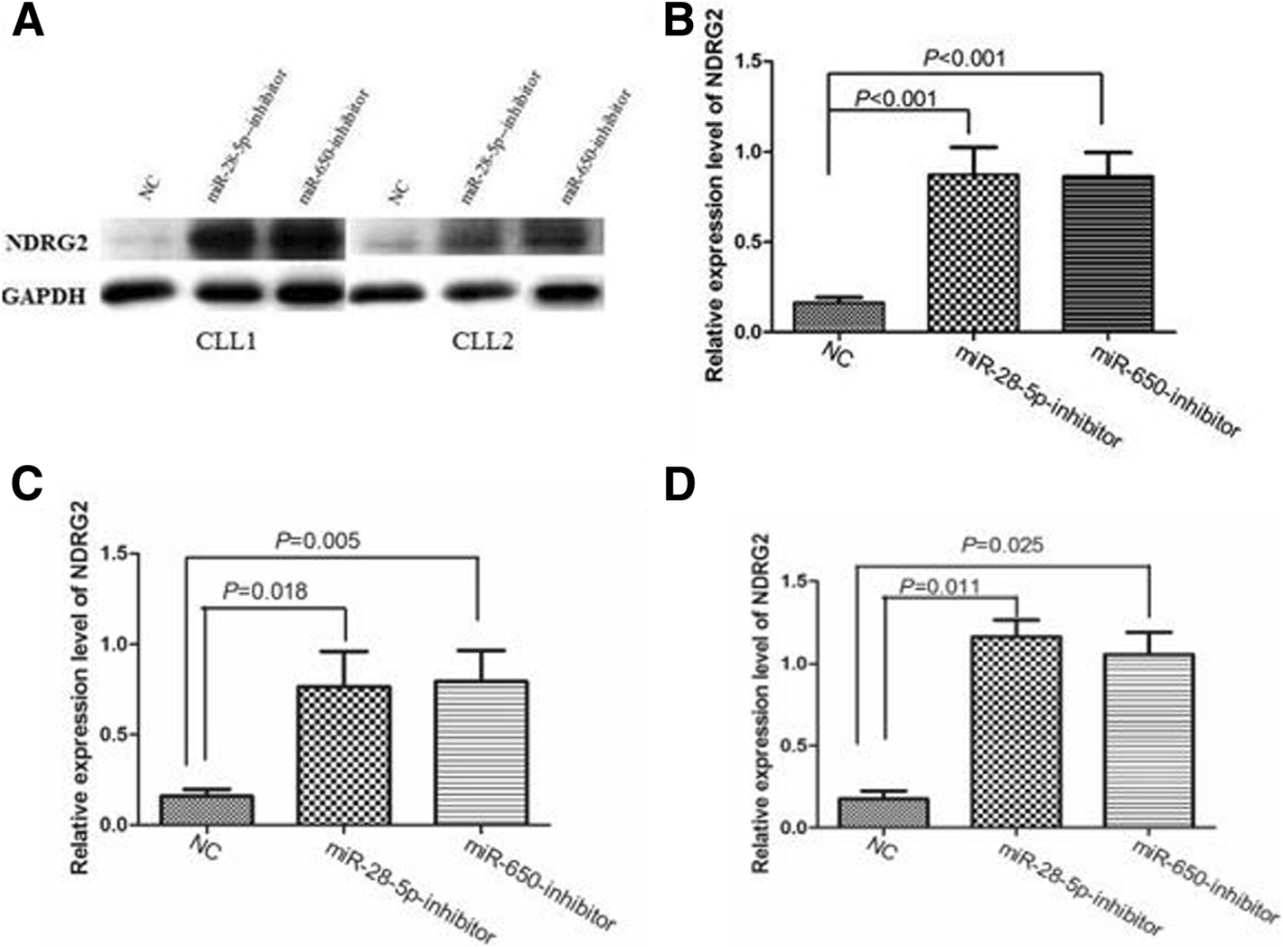
**a** Up-regulate *NDRG2* protein expression *NDRG2* protein levels after transfection with miRNA inhibitors (including miR-28-5p, miR-650 and NC miRNA inhibitors) in 2 primary CLL cells (Western blot analysis) (CLL1: CLL cells from a patient with p53 deletion; CLL2: CLL cells from a patient without p53 aberrations); **b** In CLL primary cells, miR-28-5p and miR-650 inhibitors treated groups show significantly increased *NDRG2* protein level compared to NC (median level, 0.8341 vs. 0.1252, *P* < 0.001; 0.7621 vs. 0.1252, *P* < 0.001). **c** Up-regulated *NDRG2* protein expression in CLL cells from patients without p53 aberrations after transfection with miR-28-5p inhibitor (*P* = 0.018) and miR-650 inhibitor (*P* = 0.005). **d** Up-regulated *NDRG2* protein expression in CLL cells from patients with p53 aberrations after transfection with miR-28-5p inhibitor (*P* = 0.011) and miR-650 inhibitor (*P* = 0.025)

In order to identify the roles of miR-28-5p and miR-650 in regulating *NDRG2* in primary CLL cells, we transiently transfected the primary cells from 11 newly diagnosed CLL cases were transiently transfected with miR-28-5p and miR-650 inhibitors and miR-NC, and detected the expression levels of miRNAs and *NDRG2* expression 24 h after transfection. The results verified by qRT-PCR analysis of CLL cells treated with the two miRNAs inhibitors showed remarkable knocking down of miR-28-5p and miR-650 expression (Fig. 3a, c, *P* = 0.009 and *P* = 0.019, respectively) as well as upregulated *NDRG2* at mRNA level after transfection of the two miRNAs inhibitors as compared with the cells transfected with miR-NC (Fig. 3b, d, *P* = 0.001 and *P* = 0.031, respectively). Western blot analysis showed that the median *NDRG2* protein expression was 0.1252 (range, 0.0438–0.3383), 0.8341 (range, 0.3575–2.0262) and 0.7621 (range, 0.3348–1.6500) in CLL cells transfected with miR-NC, miR-28-5p inhibitors and miR-650 inhibitors, respectively. The results indicated that *NDRG2* protein expression was markedly up-regulated after transfection with miR-28-5p inhibitors (*P* < 0.001) and miR-650 inhibitors (*P* < 0.001) compare with miR-NC (Fig. 4a, b). Moreover, up-regulated *NDRG2* protein expression was observed in CLL cells from patients without p53 aberrations (*P* < 0.05) (Fig. 4c) and with harbored p53 aberrations (*P* < 0.05) (Fig. 4d).

**Fig. 5:**
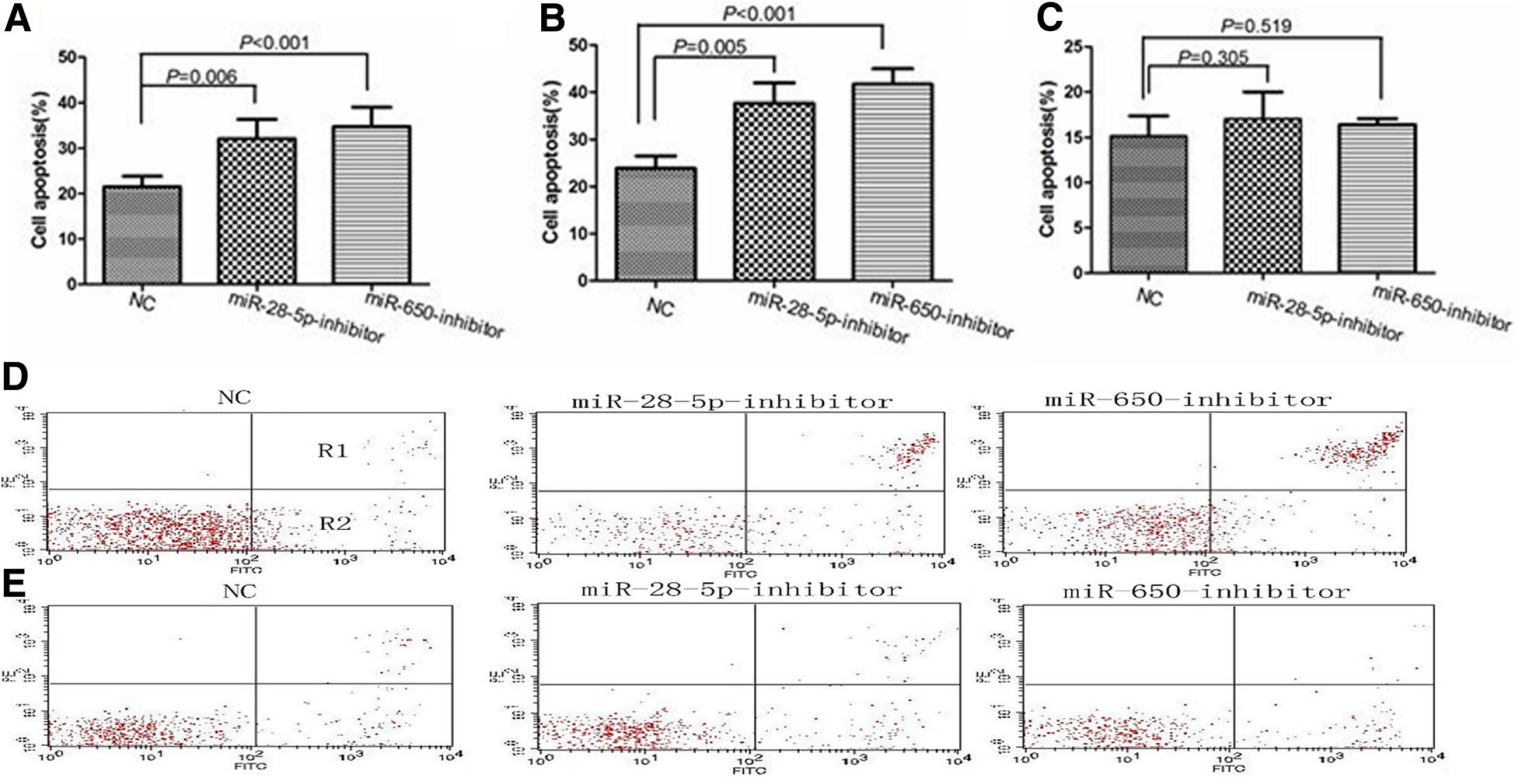
**a** miR-28-5p and miR-650 inhibitors treated groups show significantly greater percentage of average apoptosis in all CLL primary cells compared to NC (32.10% vs. 21.53%, *P* = 0.006; 34.79% vs. 21.53%, *P* < 0.001). **b** miR-28-5p and miR-650 inhibitors treated groups also show greater percentage of average apoptosis compared to NC in CLL primary cells without p53 aberrations (37.75% vs. 23.97%, *P* = 0.005; 41.68% vs. 23.97%, *P* < 0.001). **c** No increase apoptosis in CLL primary cells with p53 aberrations was observed in miR-28-5p and miR-650 inhibitors treated groups compared to NC (17.05% vs.15.16%, *P* = 0.305, 16.43% vs. 15.15%, *P* = 0.519). **d** Higher apoptosis percentage (R1 + R2) was seen in primary CLL cells without p53 aberrations treated with miR-28-5p- (41.32% vs. 19.05%) and miR-650- (41.09% vs. 19.05%) inhibitors groups than the apoptosis percentage of cells treated with miR-NC. **e** No increased apoptosis rates were found in primary CLL harboring p53 aberrations with miR-28-5p- (16.36% vs. 16.58%) and miR-650- (16.26% vs. 16.58%) inhibitors groups compared to the apoptosis rates treated with miR-NC. Y-axis: number of cells stained with PI; X-axis: cells stained with Annexin V–FITC. Apoptotic cells are in R1 and R2

Apoptosis assay was performed to ascertain the biological effect of these miRNAs inhibitors on the apoptosis of primary CLL cells. Marked increase in apoptosis was observed the primary CLL cells transfected with miR-28-5p- and miR-650-inhibitors (*P* = 0.006 and *P* < 0.001, respectively (Fig. 5a), compared to miR-NC- transfected CLL cells. Significant increase in apoptosis was also found in transfection of the two miRNAs inhibitors into CLL cells from patients without p53 aberrations compared with miR-NC (*P* = 0.005 and *P* < 0.001, respectively, Fig. 5b, d). However, no increased apoptosis rates were found in CLL with p53 aberrations, after transfecting with the above miRNAs inhibitors (*P* = 0.305 and *P* = 0.519, respectively, Fig. 5c, e). These findings demonstrated that inhibition of miR-28-5p or miR-650 could induce more apoptosis in CLL cells in p53-dependent manner.

## Discussion

*NDRG2*, located at chromosome 14q11.2, is an important member of the NDRG family and recognized as tumor suppressor. Series of studies have been done to explore the expression and the clinical significance of *NDRG2* in cancers including hematological malignancies [5, 6, 9]. Nakahata S et al. [6] found that *NDRG2* expression was significantly reduced in adult T-cell leukemia lymphoma (ATLL) cell lines and primary acute-type ATLL samples, and downregulation of *NDRG2* can activate the PI3K-AKT signaling pathway. Lchikawa T et al. [9] reported that loss of *NDRG2* also enhanced activation of the NF-κB pathway in ATLL. In current work, we tentatively investigated the expression level of *NDRG2* and its relation with prognostic factors of CLL and found marked decrease of *NDRG2* mRNA expression in CLL patients compared to HC. The observations showed that *NDRG2* was significantly downregulated in CLL patients with Binet B/C, high LDH level, IGHV un-mutated and p53 aberrations. TP53 mutation and p53 deletion, which are the powerful prognostic factors in CLL, were defined as “p53 aberrations” in this study. The result revealed that patients with lower level of *NDRG2* presented aggressive characteristics and *NDRG2* may play a critical role in the pathogenesis and development of CLL. Importantly, we found a strong correlation between *NDRG2* expression and TTT as well as OS. Patients with a lower expression level of *NDRG2* had a significantly shorter TTT and inferior OS than those with a higher *NDRG2* expression by univariate analysis. Multivariate analysis further demonstrated that *NDRG2* mRNA was prognostic value for TTT and OS independent of IGHV mutation status as well as p53 abnormalities. These findings proved that *NDRG2* expression should be a new prognostic factor for CLL. FISH analysis failed to detect del(11q22.3), del(13q14) and + 12 in part of CLL patients, for which those factors were not included in our survival analysis.

Based on the above results, we further investigated the molecular mechanisms underlying *NDRG2* regulatory pathways. MiRNAs are a family of approximate 22 nucleotides small and single-stranded non-coding RNAs that negatively regulate gene expression [12, 13]. In recent years, there is growing attention to the role of miRNAs in cancers, and abnormal miRNAs expression is extensively reported in hematologic neoplasms [14–17]. MiRNAs affect the stability of targeted oncogenes or tumor suppressors, thus leading to the impact on cellular physiology in certain malignancies. However, limited studies have ever examined miRNAs targeting *NDRG2* in CLL. Therefore, we investigated miRNAs targeting *NDRG2* and evaluate the effect on CLL cell apoptosis. Using computational analyses, four conserved miRNAs were identified as *NDRG2*-related miRNAs. The dual-luciferase assay confirmed that the mimics of miR-28-5p and miR-650 obviously suppressed the activity of *NDRG2*. In order to understand whether the *NDRG2* is a direct target of miR-28-5p and miR-650 in CLL, qRT-PCR was performed to determine the expression of *NDRG2*, miR-28-5p and miR-650 in 30 CLL patients and 10 HC. Interestingly, we found that the expression level of miR-28-5p and miR-650 were significantly increased in CLL patients compared to HC, and were negatively associated with *NDRG2*. Furthermore, we examined the effects of antisense oligonucleotides targeting miR-28-5p and miR-650 on the primary CLL cells. We investigate transfection efficiency and found that the expression of miR-28-5p and miR-650 were significantly under-expressed after transfection with those miRNA inhibitors, yet the levels of *NDRG2* mRNA and proteins were up-regulated in transfected cells with miR-28-5p and miR-650 inhibitors compared with the NC. This observation supplied strong evidence that *NDRG2* expression can be regulated by miR-28-5p and miR-650 in CLL. In other words, miR-28-5p and miR-650 can function as oncogene negatively regulating the expression of tumor suppressor in CLL.

Previous studies indicated significantly increased miR-650 expression and its association with the progression in gastric cancer, colorectal cancer, hepatocellular cancer and glioma [10, 18–20]. Further, Feng et al. [10] described regulation of *NDRG2* by miR-650 in human colorectal cancer cells, which is consistent with our findings. However, Mraz M et al. [21] found that CLL patients with higher expression of miR-650 had significantly longer OS and TTT as well as transfection with miR-650 resulted in a reduction in the proliferative capacity of B cells, which partly argued against our results. This disparity can be associated with the diverse biological characteristics of CLL in Chinese population from those of European origins. Almeida et al. [22] reported that miR-28-5p was downregulated in colorectal cancer cells compared with normal colon samples, and that overexpression of miR-28-5p reduced colorectal cancer cell proliferation, migration and invasion in vitro. This discrepancy from ours may be associated with miR-28-5p exerting different functions in diverse types of tumors. In addition, it is worth mentioning that the level of *NDRG2* mRNA and protein were both up-regulated in transfected cells with miR-28-5p and miR-650 inhibitors, which is consistent with our previous work that demonstrated up-regulated levels of PTEN mRNA and proteins in transfected cells with miR-26a and miR-214 inhibitors as compared with the controls [23]. It is generally accepted that most animal miRNAs exert their regulatory effects through incomplete matching with 3′-untranslated region (3’-UTR) of their mRNA targets, repress target-gene expression at the level of translation, reducing the protein levels of their target genes, yet the mRNA levels of these genes are barely affected [24]. Nevertheless, some findings indicate that miRNAs share only partial complementarity with their targets, and can also induce mRNA degradation and reduce the mRNA levels of their target genes in mammals [25, 26], which may explain the results in current study.

We observed the knockdown of *NDRG2* with miR-28-5p and miR-650 inhibitors inducing CLL cell apoptosis, yet found no increased apoptosis rates in patients with p53 aberrations following transfection with the above miRNAs inhibitors. Contrarily, significant apoptosis increase was seen in patients without p53 aberrations after transfection. Previous studies showed that overexpression of *NDRG2* markedly promoted tumor cell apoptosis in renal cell carcinoma [27], esophageal carcinoma [28] and breast cancer [29]. Although we found the similar results, yet overexpression of *NDRG2* promoting CLL cell apoptosis was only seen in patients without p53 aberrations. Liu et al. studied the association of *NDRG2* with p53, and reported that *NDRG2* was p53-inducible target gene that is transactivated by p53 and is required for the full p53-mediated apoptotic response [7]. Cao et al. also found that adenoviruses carrying *NDRG2* enhanced p53-mediated apoptosis of hepatocarcinoma cells [30]. In our study, we found that *NDRG2* expression was significantly reduced in CLL patients with p53 aberrations, yet increased by transfecting with related miRNAs-inhibitors promoting CLL cells apoptosis without p53 aberrations. However, association between *NDRG2* and p53 pathway in CLL needs verification by expanding population samples due to limited cases included in current work.

## Conclusions

This study for the first time has revealed that *NDRG2* expression can be down-regulated in patients with CLL, and that *NDRG2* mRNA levels may be a useful prognostic variable in CLL patients. Our results further indicate that downregulation of *NDRG2* expression is related to the aberrant expression of miR-28-5p and miR-650, and the apoptosis-inducing effects of *NDRG2* should involve p53 activation in patients with CLL. Potential therapeutic applications in CLL by restoring the *NDRG2* activity via knockdown of miR-28-5p and/or miR-650 may be considerations together with the *NDRG2*/p53 feedback loop in future work.

## Additional files

Additional file 1: Sequences of qRT-pCR primers of *NDRG2* and miRNAs. (PDF 140 kb)

Additional file 2: Predicting the possible binding sites of 4 miRNAs targeting *NDRG2* by bioinformatics. (PDF 59 kb)

Additional file 3: *NDRG2* mRNA levels indicating an inverse correlation with miR-28-5p and miR-650. (PDF 107 kb)

## Abbreviations

ALC: Absolute lymphocyte count
ATLL: Adult T-cell leukemia lymphoma
CLL: Chronic lymphocytic leukemia
FISH: Fluorescence in situ hybridization
HC: Healthy controls
IWCLL: International workshop on chronic lymphocytic leukemia
LDH: Lactate dehydrogenase
miRNAs: microRNAs
*NDRG2*: NMYC downstream-regulated gene-2
OS: Overall survival
PI: Propidium iodide
TTT: Time to first treatment
β2-MG: β2-microglobulin
