# Supplementary material for: NDRG2 mRNA levels and miR-28-5p and miR-650 activity in chronic lymphocytic leukemia
Source: BMC Cancer. 2018 Oct 22;18:1009. doi: 10.1186/s12885-018-4915-3 (PMC6196416; doi:10.1186/s12885-018-4915-3)
Supplement: Supplementary file 1 — Sequences of qRT-pCR primers of NDRG2 and miRNAs. (PDF 140 kb) [file 12885_2018_4915_MOESM1_ESM.pdf]

Additional file 1:

Sequences of qRT-pCR primers of *NDRG2* and miRNAs

**Gene**		**Primer sequence (5'-3')**
**NDRG2**	forward	CCCTGTGTTCCCTTTGGGAT
	reverse	GTGAGGCCTGTTAGCTTGTG
**β-actin**	forward	GGCGGCACCACCATGTACCCT
	reverse	AGGGGCCGGACTCGTCATACT
**U6**	RT	GTCGTATCCAGTGCAGGGTCCGAGGTATTCGCACTGGAT
	primer	ACGACAAAATA
	Forward	CTCGCTTCGGCAGCACATA
	Reverse	GTGCAGGGTCCGAGGT
**miR-28-5p**	RT	CTCAACTGGTGTCGTGGAGTCGGCAATTCAGTTGAGCTC
	primer	AATAG
	Forward	ACACTCCAGCTGGGAAGGAGCTCACAGTCT
	Reverse	TGGTGTCGTGGAGTCG
**miR-650**	RT	GTCGTATCCAGTGCGTGTCGTGGAGTCGGCAATTGCACT
	primer	GGATACGACGTCCTG
	Forward	AGAGGAGGCAGCGCTCT
	Reverse	CAGTGCGTGTCGTGGAGT
