# Supplementary material for: NDRG2 mRNA levels and miR-28-5p and miR-650 activity in chronic lymphocytic leukemia
Source: BMC Cancer. 2018 Oct 22;18:1009. doi: 10.1186/s12885-018-4915-3 (PMC6196416; doi:10.1186/s12885-018-4915-3)
Supplement: Supplementary file 2 — Predicting the possible binding sites of 4 miRNAs targeting NDRG2 by bioinformatics. (PDF 59 kb) [file 12885_2018_4915_MOESM2_ESM.pdf]

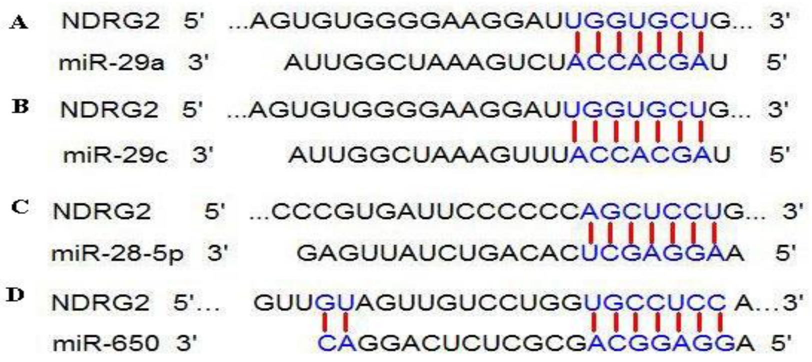

Additional file 2 Predicting the possible binding sites of 4 miRNAs targeting *NDRG2* by bioinformatics.
