# Supplementary figures and images for: NDRG2 mRNA levels and miR-28-5p and miR-650 activity in chronic lymphocytic leukemia
Source: BMC Cancer. 2018 Oct 22;18:1009. doi: 10.1186/s12885-018-4915-3 (PMC6196416; doi:10.1186/s12885-018-4915-3)

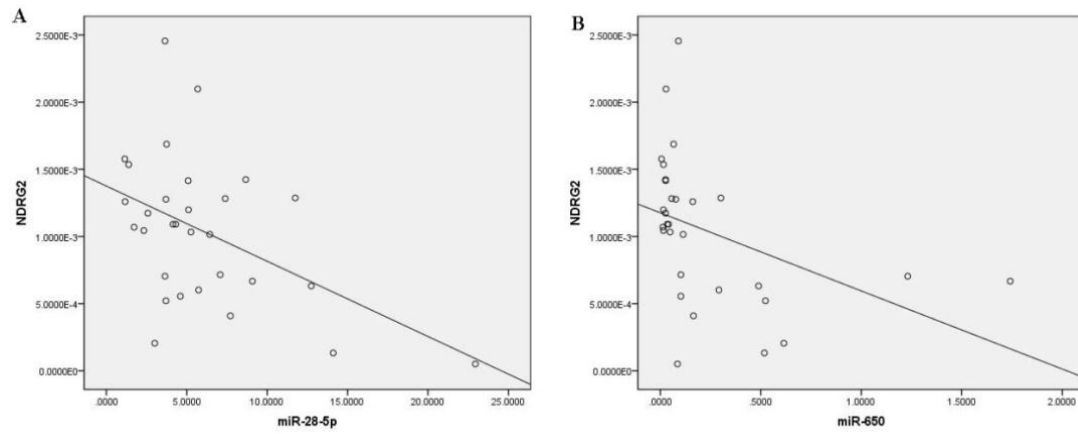

**Additional file 3** *NDRG2* mRNA levels indicating an inverse correlation with miR-28-5p and miR-650.

Supplement: Supplementary file 3 — NDRG2 mRNA levels indicating an inverse correlation with miR-28-5p and miR-650. (PDF 107 kb) [file 12885_2018_4915_MOESM3_ESM.pdf]
